# Diet Quality and Nutrition Behavior of Federal Nutrition Education Program Participants before and during the COVID-19 Pandemic

**DOI:** 10.3390/nu15010141

**Published:** 2022-12-28

**Authors:** Kavitha Sankavaram, Annie J. Roe, Jolene Whiteley, William J. Price

**Affiliations:** 1 Department of Nutrition and Food Science, University of Maryland, College Park, MD 20742, USA; 2 Margaret Ritchie School of Family and Consumer Sciences, University of Idaho, Moscow, ID 83844, USA; 3 Statistical Programs, College of Agricultural and Life Sciences, University of Idaho, Moscow, ID 83844, USA

**Keywords:** nutrition, education, EFNEP, low-income, COVID-19, food, diet quality

## Abstract

Despite challenges due to the COVID-19 pandemic, reports from regional and national meetings of the Expanded Food and Nutrition Education program (EFNEP) have provided anecdotal evidence that the program has persevered, pivoted, and continued to positively impact the lives of some of the nation’s most vulnerable populations. However, there have been necessary changes to program delivery, inevitable changes in the lives of participants, and changes in the food environment that may have impacted program outcomes. This study compares national EFNEP data (demographics, behavior change data, and 24 h dietary recall data) of participants from two federal fiscal years, before the COVID-19 pandemic and during the pandemic. Linear mixed model analysis of variance and covariance were used to assess the effects of year on program outcomes. Results of this study provide quantitative evidence of the resiliency of EFNEP to facilitate positive behavior changes related to diet quality, physical activity, food safety, food resource management, and food security. Amidst changes in the food environment during the COVID-19 pandemic, these results emphasize the importance and value of federal nutrition education programs in any food environment.

## 1. Introduction

The COVID-19 pandemic has greatly affected the health, economic, and socio-cultural conditions in the United States (USA) and around the world [1]. While mitigation strategies through local and state mandates (e.g., stay-at-home measures, quarantine, shutdowns) helped minimize viral spread, these approaches have triggered shifts in the food environment and impacted population-wide dietary practices [2]. The early stages of the pandemic (March 2020) resulted in massive unemployment and also altered food consumption behaviors such as consuming non-perishable, low-cost food [3] and online grocery shopping [4]. Nearly 44% of low-income Americans were estimated to experience food insecurity [5] and the need to obtain provisions from emergency food distribution centers [6].

Food insecurity is the inability to provide access to nutritionally adequate food for one or more household members because of little money or other resources. Food insecurity is one of the most significant public health concerns in the United States that contributes to poor diet quality and other health disparities [7]. Food insecure populations tend to decrease their consumption of fruits, vegetables, and whole grains and may replace them with calorie-dense and highly processed foods resulting in high-fat, low fiber dietary patterns [8]. The exacerbation of COVID-19 with an already stressed food security system adds further vulnerabilities to food security and diet quality, leaving individuals and families at a higher risk for diet-related chronic diseases such as obesity, diabetes, and malnutrition [9,10]. The association between food insecurity and diet-related chronic disease suggests a potential syndemic as limited food access and availability can compromise diets and healthy living behaviors, contributing to the development of chronic diseases [10].

Often, nutrition education is inadequate in underprivileged populations [11], which may further contribute to poor dietary choices [12], leading to unmet nutritional needs of family members, particularly children. Studies have shown that even households that have challenges with affording food can have better dietary quality and improved health outcomes [13] with changes in food-related behaviors, including enhanced resource management skills and nutrition practices. Nutrition education is a potential, albeit limited, solution associated with behavior change [14].

The federal policy response included several relief measures to mitigate the adverse effects of the COVID-19 crisis, including food security. Measures included expansion of many existing USA Department of Agriculture (USDA) programs and rapid development of new programming, such as the Pandemic Electronic Benefit Transfer program [15]. Flexibilities were also offered to some federal nutrition education programs [15], such as the Expanded Food and Nutrition Education Program (EFNEP), enabling online teaching methods to reach disadvantaged populations.

EFNEP is a federal extension program, conducted through 76 land grant universities, available in all USA states, territories, and the District of Columbia. Peer educators (paraprofessionals) provide nutrition education to low-income youth and adults through a series of lessons. Paraprofessionals typically live in the community they work in, meeting people where they are at. Similar in function to a Community Health Worker, paraprofessionals implement programming to promote overall community health. These individuals provide education and do not provide other health-related preventative services. EFNEP programming works to reduce nutrition-insecurity with hands-on learning that is backed by evidence and program data [16].

In this study, we comprehensively analyze change in behaviors related to diet quality, food resource management, physical activity, food safety, and food security, as well as change in diet quality score of a nationally representative sample of low-income adults participating in EFNEP before and during the COVID pandemic. We also examine whether the changes in behavioral outcomes remained the same regardless of the food environment impacting food security status and the program delivery. Programming was delivered primarily in-person before the COVID pandemic and via hybrid approaches (in-person and virtual) during the COVID pandemic. We hypothesize that changes in behaviors and diet quality scores assessed in EFNEP will be different during the COVID-19 pandemic compared to pre-pandemic.

## 2. Materials and Methods

### 2.1. Study Design and Population

This study was a secondary analysis of data collected from EFNEP participants by EFNEP paraprofessionals from two federal fiscal years (FFY) 2019 and 2020 (October 2018 through September 2020). This data set included information on all EFNEP participants across 50 states and 6 US territories. Data were entered through EFNEP’s web-based nutrition education evaluation and reporting system (WebNEERS) and submitted to the National Institute of Food and Agriculture (NIFA). These data were obtained through a freedom of information act request to NIFA. Since researchers in this study utilized deidentified data, institutional review board approval was not required as per USA Department of Health and Human Services guidelines [17].

For analysis purposes we included only those participants who were 18 to 80 years old, resided in households of 15 or fewer people, had completed between 3 and 24 lessons, and had graduated from the EFNEP program. EFNEP graduates are defined as program participants who complete a required number of lessons as well as both the entry and exit surveys [18]. Only those participants with no missing information for any of the variables were included in the study. Analyses were limited to participants who had at least one child or were pregnant, as this is the primary audience of EFNEP (adults with children).

### 2.2. Measures

Participants completed a set of questionnaires—an enrollment form, a 24 h die-tary recall (24 HDR), and the Food and Physical Activity Questionnaire (FPAQ) at the be-ginning of the program (pretest) and a replicate set of forms at the end of the program (posttest). The instructor recorded the number of lessons, sessions, and hours of education completed by each participant. Lessons refer to the individual content sections of the curricula, sessions refer to the class periods, and hours refer to the time spent receiving instruction. All data was entered into WebNEERS.

#### 2.2.1. Participant and Household Characteristics/Demographics

Data on demographics were collected through the enrollment form. Participants self-reported demographic information such as age, race, and ethnicity, place of residence, income level, and pregnancy status. Participants also reported the number and age of children in the home, number of other adults in the household, place of residence, and participation in public assistance programs such as Special Supplemental Nutrition Program for Women, Infants, and Children (WIC) and The Emergency Food Assistance Program (TEFAP).

#### 2.2.2. Food and Physical Activity Knowledge and Behaviors

Data from a validated pretest-posttest Food and Physical Activity Questionnaire (FPAQ) [19] composed of 20 core questions to evaluate behaviors was also utilized. Improvement was measured by a positive change from pretest to posttest responses. FPAQ consisted of 5 domains: (1) diet quality, (2) physical activity, (3) food safety, (4) food security, and (5) food resource management. The response options for each question were based on a Likert scale and can be seen in Supplementary Figure S1. Each question was scored with higher scores indicating greater frequency of the behavior in question. Questions on diet quality indicators included frequency of eating fruits (Q1; 1–6 points), vegetables (Q2; 1–6 points), red and orange vegetables (Q3; 1–7 points), dark green vegetables (Q4; 1–7 points), drinking regular soda (not diet) (Q5; 1–7 points), drinking fruit punch, fruit drinks, sweet tea, or sports drinks (Q6; 1–7 points), and cooking dinner at home (Q16; 1–7 points). Physical activity behaviors include exercising for at least 30 min (Q7; 1–8 points), doing workouts to build and strengthen muscles (Q8; 1–8 points), or making small changes to be more active (Q9; 1–6 points). Food safety practices comprised of questions on washing hands before preparing food (Q10; 1–6 points), washing all items and sur-faces after cutting raw meat or seafood (Q11; 1–6 points), not thawing frozen food at room temperature (Q12; 1–6 points), or using a meat thermometer (Q13; 1–6 points). Food security indicators included not eating less than you wanted so there was more food for your family (Q14; 1–6 points) or having enough money to get food for your family (Q15; 1–6 points). Food resource management practices covered cooking dinner at home (Q16; 1–6 points), comparing food prices (Q17; 1–6 points), planning meals before shopping (Q18; 1–6 points), looking in refrigerator or cupboard before shopping (Q19; 1–6 points), or making a list before shopping (Q20; 1–6 points). Cooking dinner at home is included under diet quality as well as food resource management.

#### 2.2.3. Dietary Assessment

The 24 h dietary recall collects food consumption and quantity over the past 24 h. The form also collects monthly food costs of a family (dollars). The 24 HDR data is used to measure diet quality in participants. One of the most common indictors of dietary quality is the Healthy Eating Index (HEI), which provides an overall picture of the types of foods people eat, their compliance with specific dietary recommendations, and the variety in their diets. In this study, we examined the effectiveness of the EFNEP intervention on over-all diet quality using HEI-2015 score. HEI-2015 evaluates how well an individual’s diet adheres to 2015–2020 Dietary Guidelines for Americans (DGA). A scoring system based on energy-adjusted food and nutrient intakes ranging from 0 to 100 is used to evaluate 13 components of food groups, with 100 being the best and high scores indicating better diet quality. The 13 components are divided into adequacy and moderation components. The adequacy component emphasizes intake of food groups and dietary elements that are desirable, and the moderation component includes consumption of food groups and dietary elements in limited quantities. Adequacy foods include total fruit (0–5 points), whole fruits (0–5 points), total vegetables (0–5 points), greens and beans (0–5 points), whole grains (0–10 points), dairy (0–10 points), total protein foods (0–5 points), seafood and plant proteins (0–5 points) and fatty acids (0–10 points). Moderation components include refined grains (0–10 points), sodium (0–10 points), added sugars (0–10 points) and saturated fats (0–10 points). The overall diet quality is indicated by the total HEI-15 score, while the component scores collectively show a pattern of diet quality. The total caloric intake does not influence the HEI-15 scores as they are standardized to a density of 1000 calories. The higher the HEI-15 score, the greater the adherence to the 2015–2020 DGA [20,21].

### 2.3. Data Collation and Statistical Analyses

The data encompassed 57 USA states and territories (see Supplementary Table S1) and included a total of 115,206 participants over FFY2019 (*n* = 43,983) and FFY2020 (*n* = 28,249). Prior to statistical analysis, the responses were summarized over participants to either averages or percentages as described below within each state/territory and year (*n* = 114).

Analysis of delivery measures (average number of sessions, lessons, and hours per participant) were fit to a linear mixed model analysis of variance with FFY (2019, 2020) as a fixed factor and state/territory as a random effect.

Analyses of improvement questions within each behavior domain of the FPAQ consisted of a linear mixed model analysis of covariance on average post-test scores assuming FFY (2019, 2020) as a fixed factor and the corresponding average pre-test score as a fixed covariate. State/territory was assumed to be a random effect.

Overall improvement was also assessed as the percentage of participants within each state/territory and year showing pre-to-post improvement on one or more of the questions within each domain of the FPAQ. These data were fit to a linear mixed model analysis of variance with year as a fixed effect and state/territory as a random effect.

Analyses of dietary recall response averages for HEI scores and food costs utilized a linear mixed model analysis of covariance assuming FFY (2019, 2020) as a fixed factor and the respective yearly pretest measure as a fixed covariate. State/territories were considered as random effects.

All statistical analyses were carried out using SAS, V9.4 (Proc Glimmix; SAS Inst Inc., Cary, NC, USA). Marginal adjusted means and differences were used to assess the effects of the year. Relevant year effects were considered at *p* ≤ 0.05.

## 3. Results

### 3.1. Population Characteristics

Table 1 shows the demographic characteristics of EFNEP participants by federal fis-cal year. Although there were 50% more participants in FFY2019 than FFY2020, demographic characteristics were similar between years.

### 3.2. Program Delivery

Table 2 shows program delivery characteristics by year. The number of educational sessions, lessons, and hours participants spent in class did not differ between FFY2019 and FFY2020.

Table 3 provides differences in food consumption and physical activity behavior changes between FFY2019 and FFY2020. Controlling for baseline scores, handwashing, cleaning surfaces, and making a grocery list increased from FFY2019 to FFY2020. The analysis for ‘frequency of eating less food so there was more food for family’ indicated a decrease in response from FFY2019 to FFY2020.

**Figure 1:**
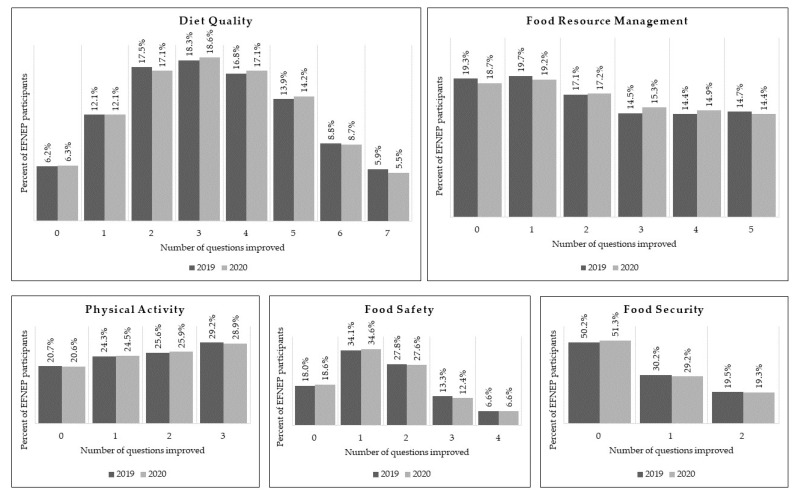
Improvement in food and physical activity related behavior scores after completing an EFNEP series. This figure shows the percentage of EFNEP participants who reported improvement in behaviors addressed in questions on the Food and Physical Activity Questionnaire related to diet quality, food resource management, physical activity, food safety, and food security.

There was no difference in the percentage of participants showing improvement in one or more questions for each domain (diet quality, *p* = 0.11; physical activity, *p* = 0.74; food safety, *p* = 0.38; food resource management, *p* = 0.99; food security, *p* = 0.94) between FFY2019 and FFY2020 as determined by ANOVA. These results are further supported when examining the distributions for the number of improved questions in each domain, where few substantial differences can be seen between FFY2019 and FFY2020 (Figure 1).

### 3.3. Healthy Eating Index and Food Cost

The analysis of overall change in HEI-15 scores indicated an increase from FFY2019 (5.56 ± 0.12) to FFY2020 (5.88 ± 0.12) (*p* = 0.04). Monthly average food costs following program participation also tended to increase from FFY2019 (352.47 ± 4.19) to FFY2020 (361.44 ± 4.19), however, it was not significant (*p* = 0.09).

## 4. Discussion

In this study, we investigated the differences in dietary behaviors, food security, and diet quality of a nationally representative sample of low-income adults completing EFNEP classes before (FFY2019) and during (FFY2020) the COVID-19 pandemic. We anticipated that COVID-19 would impact behaviors assessed in EFNEP. What we found, however, was evidence of the resiliency of this long-standing program. Although there was a decrease in EFNEP participation in FFY2020 compared to FFY2019, the percentage of participants reporting improvement in questions on the food and physical activity questionnaire was not different between years.

There were about 50% more adults participating in EFNEP in FFY2019 compared to FFY2020. This was not unexpected as the COVID-19 pandemic created an environment of social distancing beginning in the second quarter of FFY2020. On 13 March 2020, the President released an emergency declaration regarding the COVID-19 pandemic [22]. By the end of May 2020, 42 states and territories had issued stay-at-home orders [23]. These policies not only affected the ability of EFNEP educators to reach the target audience, but the overall food environment was also greatly impacted by COVID-19.

Uncertainty and anxiety around food access and safety of food may have contributed to the higher postscores seen in FFY2020 (vs. FFY2019) on some of the food safety and food resource management behaviors assessed in EFNEP. Participants reported a higher frequency of handwashing and cleaning surfaces in FFY2020 compared to FFY2019. Participants were likely receiving handwashing and surface cleaning information from multiple sources in addition to EFNEP [24]. Further, the threat of contracting COVID-19 may have provided additional motivation for behavior change. Little was known about the transmission of COVID-19, shoppers were wiping down groceries and take-out meals before consuming or storing [25].

Participants more often reported looking at what they had on-hand or making a list before shopping in FFY2020 compared to FFY2019. Several aspects of the food environment at the time may have contributed to increased motivation or necessity to change these behaviors. Out of concern of safety and following the USA Centers for Disease Control and Prevention (CDC) guidelines, many shoppers transitioned to purchasing groceries online [26]. In May of 2020, the USA Secretary of Agriculture announced that 13 new states would soon be able to purchase food online with their SNAP benefits [27]. This allowed SNAP families in 13 states and the District of Columbia to purchase food online. Grocery stores themselves became a stress as customers and workers navigated balancing the need for purchasing food with the risk of COVID-19 transmission in close quarters [28]. Retailers across the nation had limited items per person as people stockpiled food and other household products [29]. Grocery store shelves became increasingly bare; it was common to see empty shelves and limited supply of everyday items such as toilet paper [30]. News outlets advised shoppers to grocery shop every 2 weeks and only go to the stores when necessary [31].

Despite this lack of stability and certainty in the food environment, EFNEP participants reported a lower frequency of eating less than they wanted to make sure there was food for their family in FFY2020 compared to FFY2019. Media urged shoppers not receiving food assistance to wait until later in the month to shop for food, allowing those receiving food assistance to get to the grocery stores at the beginning of the month before the shelves were empty [32]. Food costs rose substantially in 2020, with at-home-food costs rising an average of 3.5% from 2019 to 2020, which was 75% above the average food price inflation observed over the last 20 years [33]. This was reflected in the data analyzed for this study. EFNEP participants reported higher monthly food costs following program participation in FFY2020 compared to FFY2019. This could also be indicative of allotting more funds to food purchases after learning about nutrition and gaining skills for cooking at home. A recent review found that individuals increased snack consumption and meal frequency during pandemic quarantine, which would also increase spending [34]. A study conducted in April 2020 found that individuals decreased frequency of shopping, but increased spending per trip [35].

Participants in FFY2020 experienced a greater change in Healthy Eating Index scores after EFNEP classes compared to participants in FFY2019. Some of this may be attributable to higher frequency of cooking at home [36]. National trends indicated that the COVID-19 pandemic resulted in a major shift toward consumers preparing and eating meals at home, with 60% saying they cooked more at home. The dietary quality of meals has also been shown to improve as the frequency of preparing and consuming meals at home increases [37]. There may also be differences in the quality of data collected in FFY2020. With a shift in the way programming was delivered, many 24 h dietary recalls were being collected via phone or one-on-one interviews rather than in group settings. This may have resulted in more detailed or accurate data collected in FY2020 compared to FY2019.

## 5. Conclusions

Despite changes in the food environment during the COVID-19 pandemic, the Expanded Food and Nutrition Education Program remained resilient and helped to improve the food and physical activity behaviors of program participants. The results of this study emphasize the importance and value of federal nutrition education programs in any food environment. The federal nutrition programs must reach to their full potential during emergencies and recession, and ensure that they are accessible, available, and adequate for people in need. Implementing evidence-based federal programs such as EFNEP may improve nutrition and reduce diet-related chronic disease outcomes in program participants. Policy makers must make it a priority to provide structural solutions and support programs that facilitate behavior change as nutrition-based food assistance programs could decrease overall healthcare spending and improve the health of children and their parents.

## Figures and Tables

**Table 1:** Expanded Food and Nutrition Education Program (EFNEP) participant demographic summary statistics in FFY2019 and FFY2020.

Characteristics	FFY2019	FFY2020
**Number of participants**	43,983	28,249
**Age (mean ± SD) (years)**	38.1 ± 12.2	38.2 ± 12.0
**Identified Sex (%)**		
Male	15	14
Female	85	86
**Ethnicity (%)**		
Hispanic	40	43
Not Hispanic	55	53
Not provided	5	4
**Race (%)**		
White	51	53
American Indian or Alaska Native	2	2
Asian	4	3
Black or African American	24	23
Native HI or Pacific Islander	2	2
Not Provided	15	15
**Education (%)**		
Middle School	9	8
High School	13	12
High School Graduate or GED	33	31
Some College	15	15
College Graduate	13	14
Post Graduate	2	2
Not Provided	15	18
**Residence (%)**		
Farm	2	1
Town with population <10,000	16	16
Town with population 10,000 to 50,000	26	25
Suburb with population >50,000	6	7
Central city with population >50,000	50	50

FFY = Federal Fiscal Year.

**Table 2:** Expanded Food and Nutrition Education Program (EFNEP) delivery characteristics in FFY2019 and FFY2020 ^a^.

	FFY2019	FFY2020	*p*
Number of sessions per participant	7.17 ± 0.21	7.10 ± 0.21	0.56
Number of lessons per participant	8.15 ± 0.20	8.01 ± 0.20	0.20
Number of hours per participant	10.60 ± 0.42	10.15 ± 0.42	0.15

^a^ Values presented as estimated mean ± standard error. FFY = Federal Fiscal Year.

**Table 3:** Food and physical activity behavior scores of Expanded Food and Nutrition Education Program (EFNEP) participants in FFY2019 and FFY2020 ^a^.

Behavior ^b^	FFY2019	FFY2020	*p*
**Diet Quality**			
(Q1) How many times a day do you eat fruit? (rarely to 4 or more times per day; 1–6 points)	3.07 ± 0.05	3.72 ± 0.05	0.60
(Q2) How many times a day do you eat vegetables? (rarely to ≥4 times per day; 1–6 points)	3.84 ± 0.05	3.85 ± 0.05	0.84
(Q3) Over the last week, how many days did you eat red and orange vegetables? (did not eat to 6–7 days per week; 1–7 points)	3.84 ± 0.05	3.88 ± 0.05	0.35
(Q4) Over the last week, how many days did you eat dark green vegetables? (did not eat to 6–7 days per week; 1–7 points)	4.08 ± 0.05	4.12 ± 0.05	0.43
(Q5) How often do you drink regular sodas (not diet)? (never to ≥4 times a day; 1–7 points)	2.18 ± 0.06	2.11 ± 0.06	0.11
(Q6) How often do you drink fruit punch, fruit drinks, sweet tea, or sports drinks? (never to ≥4 times a day; 1–7 points)	2.27 ± 0.06	2.22 ± 0.06	0.25
(Q16) How many days a week do you cook dinner (your main meal) at home? (rarely to 6–7 days a week; 1–7 points)	5.57 ± 0.05	5.64 ± 0.05	0.11
**Physical Activity**			
(Q7) In the past week, how many days did you exercise for at least 30 min? (0 to 7 days; 1–8 points)	4.31 ± 0.06	4.34 ± 0.06	0.62
(Q8) In the past week, how many days did you do workouts to build and strengthen your muscles? (0 to 7 days; 1–8 points)	3.23 ± 0.07	3.26 ± 0.07	0.58
(Q9) How often do you make small changes on purpose to be more active? (never to always; 1–6 points)	3.64 ± 0.05	3.70 ± 0.05	0.12
**Food Safety**			
(Q10) How often do you wash your hands with soap and running water before preparing food? (never to always; 1–6 points)	5.67 ± 0.02	5.72 ± 0.02	0.01
(Q11) After cutting raw meat or seafood, how often do you wash all items and surfaces that came in contact with these foods? (never to always; 1–6 points)	5.63 ± 0.02	5.69 ± 0.02	0.01
(Q12) How often do you thaw frozen food on the counter or in the sink at room temperature? (never to always; 1–6 points)	2.84 ± 0.09	2.79 ± 0.09	0.27
(Q13) How often do you use a meat thermometer to see if meat is cooked to a safe temperature? (never to always; 1–6 points)	2.98 ± 0.08	3.02 ± 0.08	0.47
**Food Resource Management**			
(Q16) How many days a week do you cook dinner (your main meal) at home? (rarely to 6–7 days a week; 1–7 points)	5.57 ± 0.05	5.64 ± 0.05	0.11
(Q17) How often do you compare food prices to save money? (never to always; 1–6 points)	4.73 ± 0.04	4.79 ± 0.04	0.08
(Q18) How often do you plan your meals before you shop for groceries? (never to always; 1–6 points)	4.40 ± 0.05	4.45 ± 0.05	0.20
(Q19) How often do you look in the refrigerator or cupboard to see what you need before you go shopping? (never to always; 1–6 points)	4.85 ± 0.04	4.91 ± 0.04	0.07
(Q20) How often do you make a list before going shopping? (never to always; 1–6 points)	4.61 ± 0.04	4.703 ± 0.04	0.01
**Food Security**			
(Q14) In the past month, how often did you eat less than you wanted so there was more food for your family? (never to always; 1–6 points)	2.33 ± 0.06	2.25 ± 0.06	0.02
(Q15) In the past month, how often did you not have money or another way to get enough food for your family (such as SNAP, WIC, or food pantry)? (never to always; 1–6 points)	1.98 ± 0.06	1.99 ± 0.06	0.88

^a^ Response options were based on a Likert scale with higher values depicting higher frequency of the behaviors in question. Values presented as estimated mean ± standard error. ^b^ Q1, Q2, etc. refers to the question number on the Food and Physical Activity Questionnaire. FFY = Federal Fiscal Year

## Data Availability

The data presented in this study are available upon request from the corresponding authors.

## Supplementary Materials

The following supporting information can be downloaded at: https://www.mdpi.com/article/10.3390/nu15010141/s1, Table S1: List of USA States and Territories Reporting EFNEP Data in FFY2019 and FFY2020; Figure S1: Food and Physical Activity Questionnaire
