# Supplementary material for: Diet Quality and Nutrition Behavior of Federal Nutrition Education Program Participants before and during the COVID-19 Pandemic
Source: Nutrients. 2022 Dec 28;15(1):141. doi: 10.3390/nu15010141 (PMC9824662; doi:10.3390/nu15010141)
Supplement: Supplementary file 1 [file nutrients-15-00141-s001.zip › nutrients-2095351-supplementary.pdf]

Supplementary Table S1. List of U.S. States and Territories Reporting EFNEP Data in FFY2019 and FFY2020

Alaska
Alabama
Arkansas
American Samoa
Arizona
California
Colorado
Connecticut
District of Columbia
Delaware
Florida
Georgia
Guam
Hawaii
Iowa
Idaho
Illinois
Indiana
Kansas
Kentucky
Louisiana
Maine
College of Micronesia
Maryland
Maine
Michigan
Minnesota
Missouri
Northern Mariana Islands
Mississippi
Montana
North Carolina
North Dakota
Nebraska
New Hampshire
New Jersey
New Mexico
Nevada
New York
Ohio

Oklahoma
Oregon
Pennsylvania
Puerto Rico
Rhode Island
South Carolina
South Dakota
Tennessee
Texas
Utah
Virginia
Virgin Islands
Vermont
Washington
Wisconsin
West Virginia
Wyoming

EFNEP= Expanded Food and Nutrition Education Program

FFY= Federal Fiscal Year

Supplementary Figure S1. Food and Physical Activity Questionnaire

☐ ENTRY ☐ EXIT

NAME

DATE / /

Note: Score is noted in parentheses following each response option. These numbers are not included on the survey provided to subjects.

Food & Physical Activity Questionnaire

Please mark the response that **best** describes how you **usually** do things.

1. How many times a day do you eat fruit?

Examples of fruits are apples, bananas, oranges, grapes, raisins, melon and berries. Include fresh, frozen, dried, or canned fruit. *Do not include juice.*

- ☐ I rarely eat fruit (1)
- ☐ Less than 1 time a day (a couple times a week) (2)
- ☐ 1 time a day (3)
- ☐ 2 times a day (4)
- ☐ 3 times a day (5)
- ☐ 4 or more times a day (6)

2. How many times a day do you eat vegetables?

Examples of vegetables are green salad, corn, green beans, carrots, potatoes, greens, and squash. Include fresh, canned and frozen vegetables. *Do not count french fries, potato chips or rice.*

- ☐ I rarely eat vegetables (1)
- ☐ Less than 1 time a day (a couple times a week) (2)
- ☐ 1 time a day (3)
- ☐ 2 times a day (4)
- ☐ 3 times a day (5)
- ☐ 4 or more times a day (6)

3. Over the last week, how many days did you eat red and orange vegetables?

Examples of red or orange vegetables are tomatoes, red peppers, carrots, sweet potatoes, winter squash, and pumpkin.

- ☐ I did not eat red and orange vegetables (1)
- ☐ 1 day a week (2)
- ☐ 2 days a week (3)
- ☐ 3 days a week (4)
- ☐ 4 days a week (5)
- ☐ 5 days a week (6)
- ☐ 6 or 7 days a week (7)

4. Over the last week, how many days did you eat dark green vegetables?

Examples of dark green vegetables are broccoli, spinach, dark green lettuce, turnip greens, or mustard greens.

- ☐ I did not eat dark green vegetables (1)
- ☐ 1 day a week (2)
- ☐ 2 days a week (3)
- ☐ 3 days a week (4)
- ☐ 4 days a week (5)
- ☐ 5 days a week (6)
- ☐ 6 or 7 days a week (7)

5. How often do you drink regular sodas (not diet)?

- ☐ Never (1)
- ☐ 1 – 3 times a week (2)
- ☐ 4 – 6 times a week (3)
- ☐ 1 time a day (4)
- ☐ 2 times a day (5)
- ☐ 3 times a day (6)
- ☐ 4 or more times a day (7)

6. How often do you drink fruit punch, fruit drinks, sweet tea or sports drinks?

- ☐ Never (1)
- ☐ 1 – 3 times a week (2)
- ☐ 4 – 6 times a week (3)
- ☐ 1 time a day (4)
- ☐ 2 times a day (5)
- ☐ 3 times a day (6)
- ☐ 4 or more times a day (7)

There is more on the next page ➧

7. In the past week, <u>how many days</u> did you exercise for at least 30 minutes?

This includes things like jogging, playing soccer, and doing fitness or dance classes, or exercise videos. <u>This 30 minutes could be all at once or 10 minutes or more at a time.</u> *Do not count housework, taking care of your kids, or walking from place to place.*

- ☐ 0 days (1)
- ☐ 1 day (2)
- ☐ 2 days (3)
- ☐ 3 days (4)
- ☐ 4 days (5)
- ☐ 5 days (6)
- ☐ 6 days (7)
- ☐ 7 days (8)

8. In the past week, <u>how many days</u> did you do workouts to build and strengthen your muscles?

This includes things like lifting weights and doing push-ups, sit-ups or planks.

- ☐ 0 days (1)
- ☐ 1 day (2)
- ☐ 2 days (3)
- ☐ 3 days (4)
- ☐ 4 days (5)
- ☐ 5 days (6)
- ☐ 6 days (7)
- ☐ 7 days (8)

9. How often do you make small changes on purpose to be more active?

This includes things like walking instead of driving, getting off the bus one stop early, doing a few minutes of exercise, or moving around instead of sitting while watching TV.

- ☐ Never (1)
- ☐ Rarely (about 20% of the time) (2)
- ☐ Sometimes (about 40% of the time) (3)
- ☐ Often (about 60% of the time) (4)
- ☐ Usually (about 80% of the time) (5)
- ☐ Always (6)

10. How often do you wash your hands with soap and running water before preparing food?

- ☐ Never (1)
- ☐ Rarely (about 20% of the time) (2)
- ☐ Sometimes (about 40% of the time) (3)
- ☐ Often (about 60% of the time) (4)
- ☐ Usually (about 80% of the time) (5)
- ☐ Always (6)

11. After cutting raw meat or seafood, how often do you wash all items and surfaces that came in contact with these foods?

- ☐ Never (1)
- ☐ Rarely (about 20% of the time) (2)
- ☐ Sometimes (about 40% of the time) (3)
- ☐ Often (about 60% of the time) (4)
- ☐ Usually (about 80% of the time) (5)
- ☐ Always (6)

12. How often do you thaw frozen food on the counter or in the sink at room temperature?

- ☐ Never (1)
- ☐ Rarely (about 20% of the time) (2)
- ☐ Sometimes (about 40% of the time) (3)
- ☐ Often (about 60% of the time) (4)
- ☐ Usually (about 80% of the time) (5)
- ☐ Always (6)

13. How often do you use a meat thermometer to see if meat is cooked to a safe temperature?

- ☐ Never (1)
- ☐ Rarely (about 20% of the time) (2)
- ☐ Sometimes (about 40% of the time) (3)
- ☐ Often (about 60% of the time) (4)
- ☐ Usually (about 80% of the time) (5)
- ☐ Always (6)

14. In the past month, how often did you eat less than you wanted so there was more food for your family?

- ☐ Never (1)
- ☐ Rarely (about 20% of the time) (2)
- ☐ Sometimes (about 40% of the time) (3)
- ☐ Often (about 60% of the time) (4)
- ☐ Usually (about 80% of the time) (5)
- ☐ Always (6)

There is more on the next page ➧

15. In the past month, how often did you not have money or another way to get enough food for your family (such as SNAP, WIC, or a food pantry)?

☐ Never (1)
☐ Rarely (about 20% of the time) (2)
☐ Sometimes (about 40% of the time) (3)
☐ Often (about 60% of the time) (4)
☐ Usually (about 80% of the time) (5)
☐ Always (6)

16. How many <u>days a week</u> do you cook dinner (your main meal) at home?

☐ I rarely cook dinner at home (1)
☐ 1 day a week (2)
☐ 2 days a week (3)
☐ 3 days a week (4)
☐ 4 days a week (5)
☐ 5 days a week (6)
☐ 6 or 7 days a week (7)

17. How often do you compare food prices to save money?

☐ Never (1)
☐ Rarely (about 20% of the time) (2)
☐ Sometimes (about 40% of the time) (3)
☐ Often (about 60% of the time) (4)
☐ Usually (about 80% of the time) (5)
☐ Always (6)

18. How often do you plan your meals before you shop for groceries?

☐ Never (1)
☐ Rarely (about 20% of the time) (2)
☐ Sometimes (about 40% of the time) (3)
☐ Often (about 60% of the time) (4)
☐ Usually (about 80% of the time) (5)
☐ Always (6)

19. How often do you look in the refrigerator or cupboard to see what you need before you go shopping?

☐ Never (1)
☐ Rarely (about 20% of the time) (2)
☐ Sometimes (about 40% of the time) (3)
☐ Often (about 60% of the time) (4)
☐ Usually (about 80% of the time) (5)
☐ Always (6)

20. How often do you make a list before going shopping?

☐ Never (1)
☐ Rarely (about 20% of the time) (2)
☐ Sometimes (about 40% of the time) (3)
☐ Often (about 60% of the time) (4)
☐ Usually (about 80% of the time) (5)
☐ Always (6)
